# Supplementary material for: Aspergillus fumigatus mitogen-activated protein kinase MpkA is involved in gliotoxin production and self-protection
Source: Nat Commun. 2024 Jan 2;15:33. doi: 10.1038/s41467-023-44329-1 (PMC10762094; doi:10.1038/s41467-023-44329-1)
Supplement: Supplementary file 3 — Description of Additional Supplementary Files [file 41467_2023_44329_MOESM3_ESM.pdf]

Description of Additional Supplementary Files

File Name: Supplementary Data S1
Description: List of proteins identified in the wild-type, GliT:GFP and GtmA:GFP upon immunoprecipitation during gliotoxin production conditions.
