# Supplementary material for: Aspergillus fumigatus mitogen-activated protein kinase MpkA is involved in gliotoxin production and self-protection
Source: Nat Commun. 2024 Jan 2;15:33. doi: 10.1038/s41467-023-44329-1 (PMC10762094; doi:10.1038/s41467-023-44329-1)
Supplement: Supplementary file 5 — Reporting Summary [file 41467_2023_44329_MOESM5_ESM.pdf]

nature portfolio

Corresponding author(s): Goldman GH

Last updated by author(s): Dec 5, 2023

Reporting Summary

Nature Portfolio wishes to improve the reproducibility of the work that we publish. This form provides structure for consistency and transparency in reporting. For further information on Nature Portfolio policies, see our Editorial Policies and the Editorial Policy Checklist.

Statistics

For all statistical analyses, confirm that the following items are present in the figure legend, table legend, main text, or Methods section.

n/a	Confirmed	
☐	☒	The exact sample size (*n*) for each experimental group/condition, given as a discrete number and unit of measurement
☒	☐	A statement on whether measurements were taken from distinct samples or whether the same sample was measured repeatedly
☐	☒	The statistical test(s) used AND whether they are one- or two-sided<br>*Only common tests should be described solely by name; describe more complex techniques in the Methods section.*
☒	☐	A description of all covariates tested
☐	☒	A description of any assumptions or corrections, such as tests of normality and adjustment for multiple comparisons
☐	☒	A full description of the statistical parameters including central tendency (e.g. means) or other basic estimates (e.g. regression coefficient) AND variation (e.g. standard deviation) or associated estimates of uncertainty (e.g. confidence intervals)
☐	☒	For null hypothesis testing, the test statistic (e.g. *F*, *t*, *r*) with confidence intervals, effect sizes, degrees of freedom and *P* value noted<br>*Give P values as exact values whenever suitable.*
☒	☐	For Bayesian analysis, information on the choice of priors and Markov chain Monte Carlo settings
☒	☐	For hierarchical and complex designs, identification of the appropriate level for tests and full reporting of outcomes
☒	☐	Estimates of effect sizes (e.g. Cohen's *d*, Pearson's *r*), indicating how they were calculated

Our web collection on statistics for biologists contains articles on many of the points above.

Software and code

Policy information about availability of computer code

Data collection	No software was used
Data analysis	The following sofwares were used: 1) Graphpad Prism 5.0 (GraphPad Software , Inc., La Jolla, CA, USA), 2) AxioVision software (version 4.8), Carl Zeiss, 3) LabSolutions software (Shimadzu Corporation, Kyoto, Japan) , and 4) Thermo Xcalibur 3.0.63 (Thermo Fisher Scientific Inc.).

For manuscripts utilizing custom algorithms or software that are central to the research but not yet described in published literature, software must be made available to editors and reviewers. We strongly encourage code deposition in a community repository (e.g. GitHub). See the Nature Portfolio guidelines for submitting code & software for further information.

Data

Policy information about availability of data

All manuscripts must include a data availability statement. This statement should provide the following information, where applicable:

- Accession codes, unique identifiers, or web links for publicly available datasets
- A description of any restrictions on data availability
- For clinical datasets or third party data, please ensure that the statement adheres to our policy

Data availability statement. All the data are available as supplementary tables and figures. Proteomic data was deposited at ProteomeExchange (https://www.proteomexchange.org) under the accession number PXD041133 (https://massive.ucsd.edu/ProteoSAFe/dataset.jsp?task=fedcc33de1ac459282153146d7585b33). Extracts from A. fumigatus mutants were identified using the GNPS database (http://gnps.ucsd.edu). The GNPS

Feature-based Molecular Networking (FBMN) analysis was performed following the protocol already established on the website (https://ccms-ucsd.github.io/GNPSDocumentation/featurebasedmolecularnetworking/). MS/MS spectra were selected with only the top five fragmentation ions in the ± 5 ppm window. The mass tolerance of precursor ions and MS/MS fragment ions were adjusted to 0.02 Da in both cases. The spectral libraries used for this study were pre-established according to the features table generated by MZMine 3 (http://mzmine.github.io/). Correspondences between network spectra and libraries were filtered to show values greater than 0.7 (Cosine Score). The results obtained for this study are available at: https://gnps.ucsd.edu/ProteoSAFe/status.jsp?task=ea7a4f8507f5433db1a6bc50fb9ca67a. The SMs data was deposited at MassIVE, Center for Computational Mass Spectrometry, full Member of the Proteome Xchange consortium. To access the data, click in the link:: https://doi.org/doi:10.25345/C5K35MQ4H. The access number MassIVE: MSV000092041. The login is MSV000092041_reviewer and the password is Gliotoxin_2023. Source data are provided with this paper.

Research involving human participants, their data, or biological material

Policy information about studies with human participants or human data. See also policy information about sex, gender (identity/presentation), and sexual orientation and race, ethnicity and racism.

Reporting on sex and gender	Our current studies do not involve human research.
Reporting on race, ethnicity, or other socially relevant groupings	Our current studies do not involve human research.
Population characteristics	Our current studies do not involve human research.
Recruitment	Our current studies do not involve human research.
Ethics oversight	Our current studies do not involve human research.

Note that full information on the approval of the study protocol must also be provided in the manuscript.

Field-specific reporting

Please select the one below that is the best fit for your research. If you are not sure, read the appropriate sections before making your selection.

☒ Life sciences ☐ Behavioural & social sciences ☐ Ecological, evolutionary & environmental sciences

For a reference copy of the document with all sections, see nature.com/documents/nr-reporting-summary-flat.pdf

Life sciences study design

All studies must disclose on these points even when the disclosure is negative.

Sample size	The sample size used in this study was usually determined based on the cost, time, or convenience of collecting the data, and the need for it to offer sufficient statistical power. The rationale for determining the sample size is based on routine experiments where at least three samples are used for microbial or growth or metabolic activity while sample size for cell lines and animal experiments was between 6 and 10 samples.
Data exclusions	No data were excluded from the analysis
Replication	All the experiments in this manuscript were repeated at least twice and most of the time, three times. All attempts of replication were successful
Randomization	All the experiments were randomized either by their position in the incubator chambers for microbial growth and metabolic activity, or in the distribution of cages in the cage incubator
Blinding	Although all the experiments were randomized, blinding was not relevant for interpreting the results of our study. There is no specific reason why to use blinding experiments for the kind of experiments we are doing.

Reporting for specific materials, systems and methods

We require information from authors about some types of materials, experimental systems and methods used in many studies. Here, indicate whether each material, system or method listed is relevant to your study. If you are not sure if a list item applies to your research, read the appropriate section before selecting a response.

Materials & experimental systems

n/a	Involved in the study	
☐	☒	Antibodies
☒	☐	Eukaryotic cell lines
☒	☐	Palaeontology and archaeology
☐	☒	Animals and other organisms
☒	☐	Clinical data
☒	☐	Dual use research of concern
☒	☐	Plants

Methods

n/a	Involved in the study	
☒	☐	ChIP-seq
☒	☐	Flow cytometry
☒	☐	MRI-based neuroimaging

Antibodies

Antibodies used

The following antibodies were used in this work: 1) 1:5,000 dilution of the mouse monoclonal GFP antibody (Santa Cruz Biotechnology), 2) secondary antibody anti-mouse IgG HRP conjugate (Cell Signaling Technology), at 1:5,000 dilution. 3) a mouse monoclonal anti-HA antibody (Sigma) at 1:5,000 dilution, as a primary antibody, 4) the same anti-mouse IgG HRP conjugate as a secondary antibody, 5) an anti-phospho p44/42 MAPK antibody (Cell Signaling Technologies) using a 1:10,000 dilution, and 6) a primary antibody was detected using an HRP-conjugated secondary antibody raised in rabbit (Sigma).

Validation

All these antibodies were validated by using negative controls from the wild-type and mutants strains, as well as positive controls for epitope tagging.

Animals and other research organisms

Policy information about studies involving animals; ARRIVE guidelines recommended for reporting animal research, and Sex and Gender in Research

Laboratory animals

Mice BALB/c, eight to nine weeks old. Cages are well ventilated, softly lit and subjected to a 12:12 light-dark cycle. The relative humidity was kept at 40 to 60%. Mouse rooms and cages were kept at a temperature range of 22oC.

Wild animals

This study did not involve wild animals

Reporting on sex

Sex was visually assigned. Females for BALB/c and males for C57BL/6

Field-collected samples

The study did not involve samples collected from the field

Ethics oversight

The principles that guide our studies are based on the Declaration of Animal Rights ratified by UNESCO on January 27, 1978 in its 8th and 14th articles. All protocols adopted in this study were approved by the local ethics committee for animal experiments from the University of São Paulo, Campus of Ribeirão Preto (Permit Number: 08.1.1277.53.6; Studies on the interaction of Aspergillus fumigatus with animals). Groups of five animals were housed in individually ventilated cages and were cared for in strict accordance with the principles outlined by the Brazilian College of Animal Experimentation (COBEA) and Guiding Principles for Research Involving Animals and Human Beings, American Physiological Society. All efforts were made to minimize suffering. Animals were clinically monitored at least twice daily and humanely sacrificed if moribund (defined by lethargy, dyspnea, hypothermia and weight loss). All stressed animals were sacrificed by cervical dislocation.

Note that full information on the approval of the study protocol must also be provided in the manuscript.

Plants

Seed stocks

No plants were used in this work.

Novel plant genotypes

No plants were used in this work.

Authentication

No plants were used in this work.
