# Supplementary material for: Aspergillus fumigatus mitogen-activated protein kinase MpkA is involved in gliotoxin production and self-protection
Source: Nat Commun. 2024 Jan 2;15:33. doi: 10.1038/s41467-023-44329-1 (PMC10762094; doi:10.1038/s41467-023-44329-1)
Supplement: Supplementary file 1 — Supplementary Information [file 41467_2023_44329_MOESM1_ESM.pdf]

Supplementary Figure S1 – *A. fumigatus* wild-type and mutant strains were grown for 5 days on MM or YAG at 37 ^{o}C.

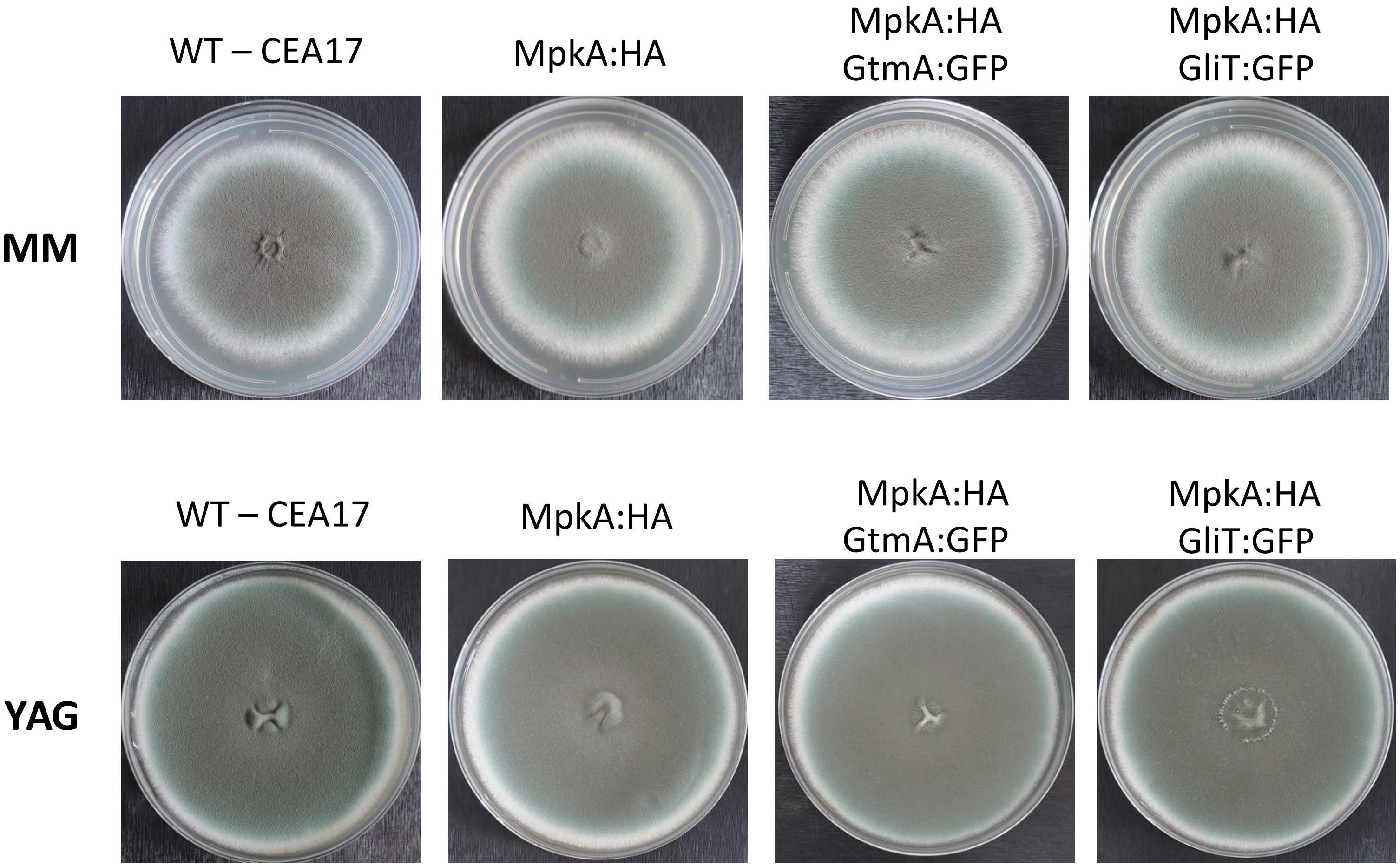

Supplementary Figure S1 - *A. fumigatus* wild-type and mutant strains were grown for 5 days on MM or YAG at 37 ^{o}C.

Supplementary Figure S2 – GliT:GFP and GtmA:GFP translocation to the vacuoles during 24 hs under gliotoxin production conditions in Czapek-dox medium.

A.

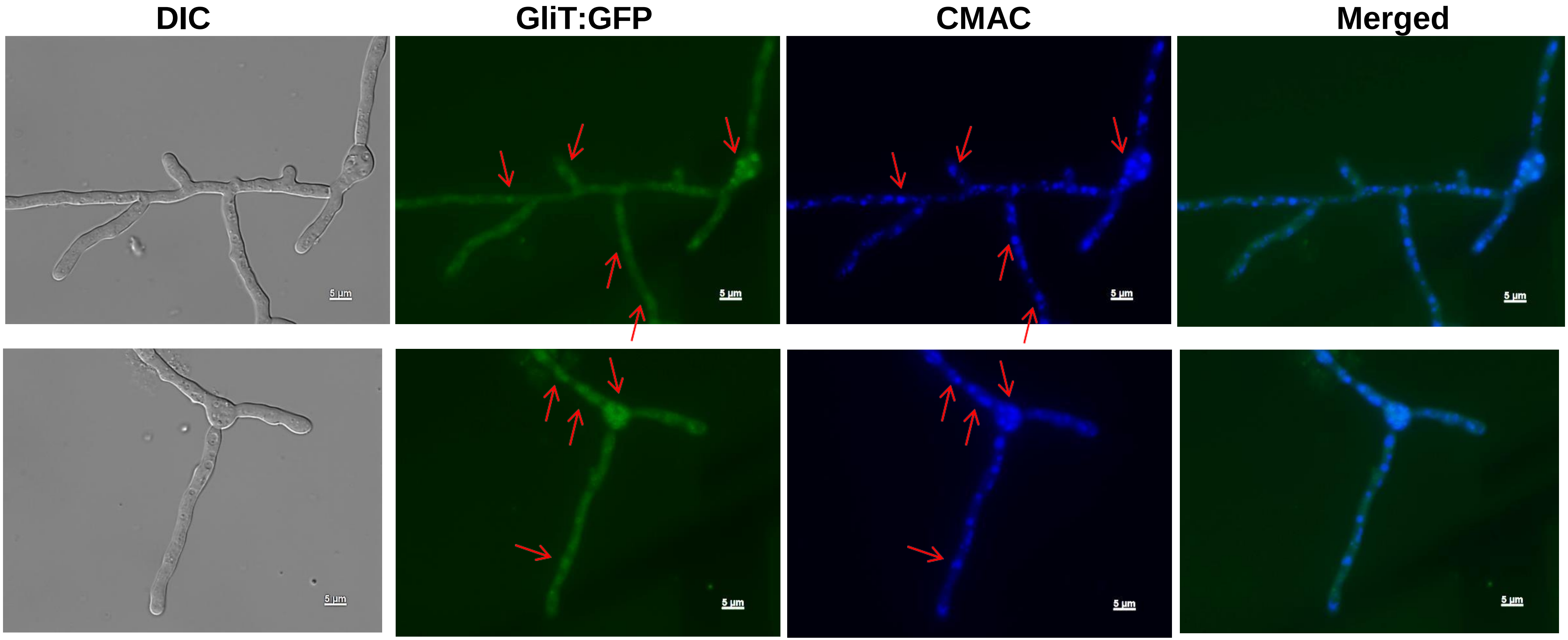

B.

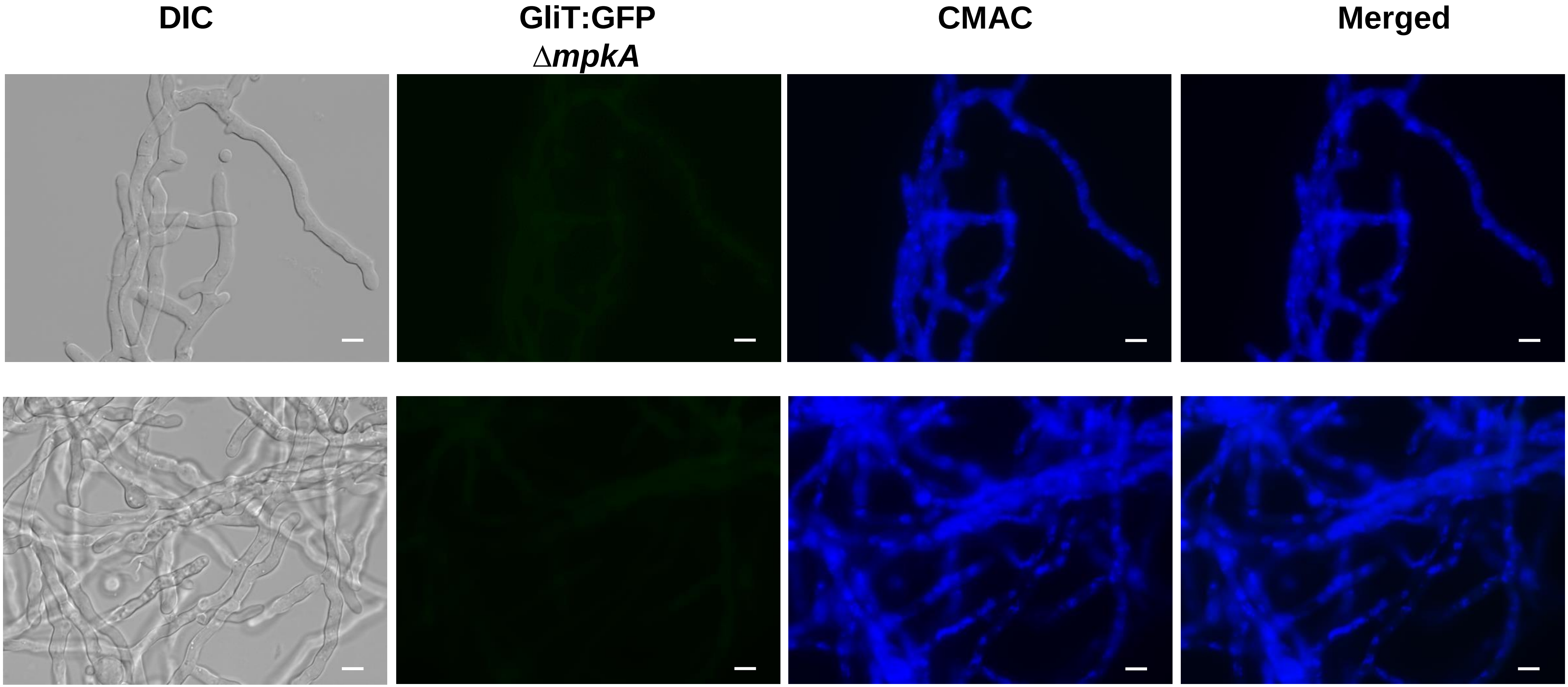

C.

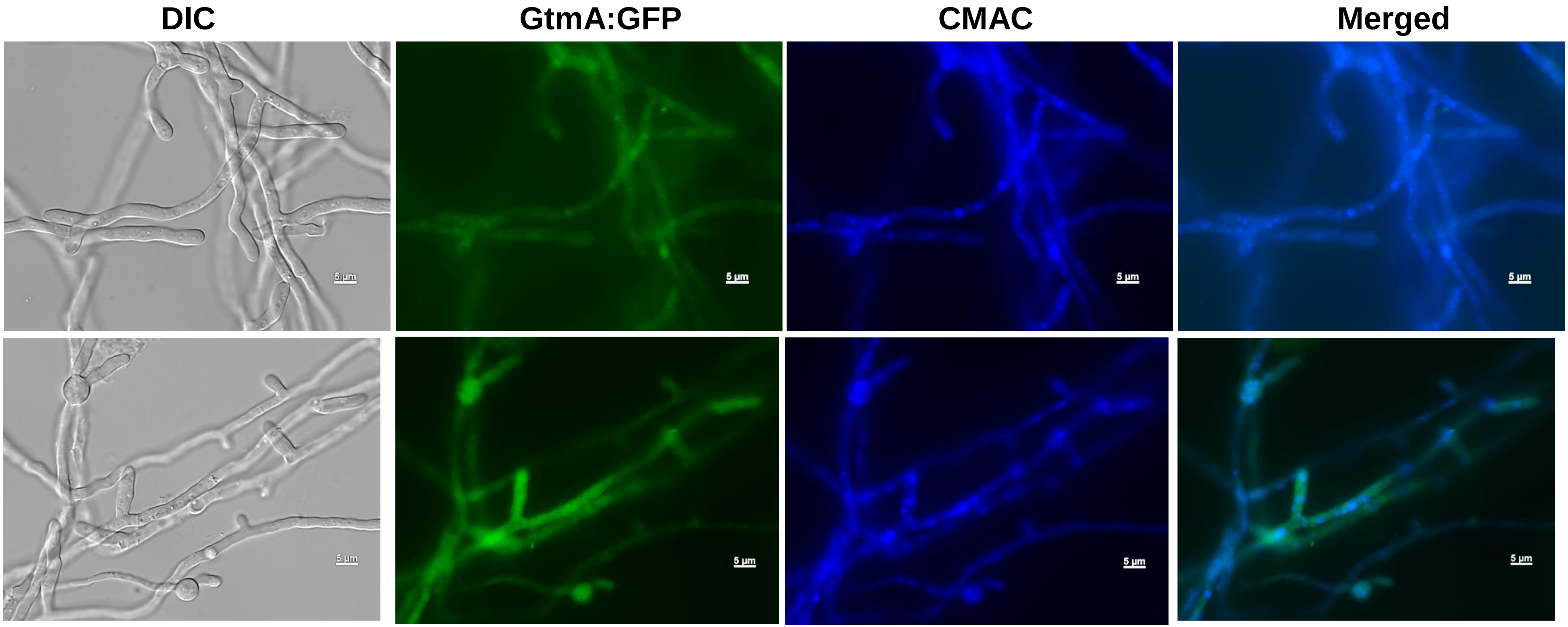

D.

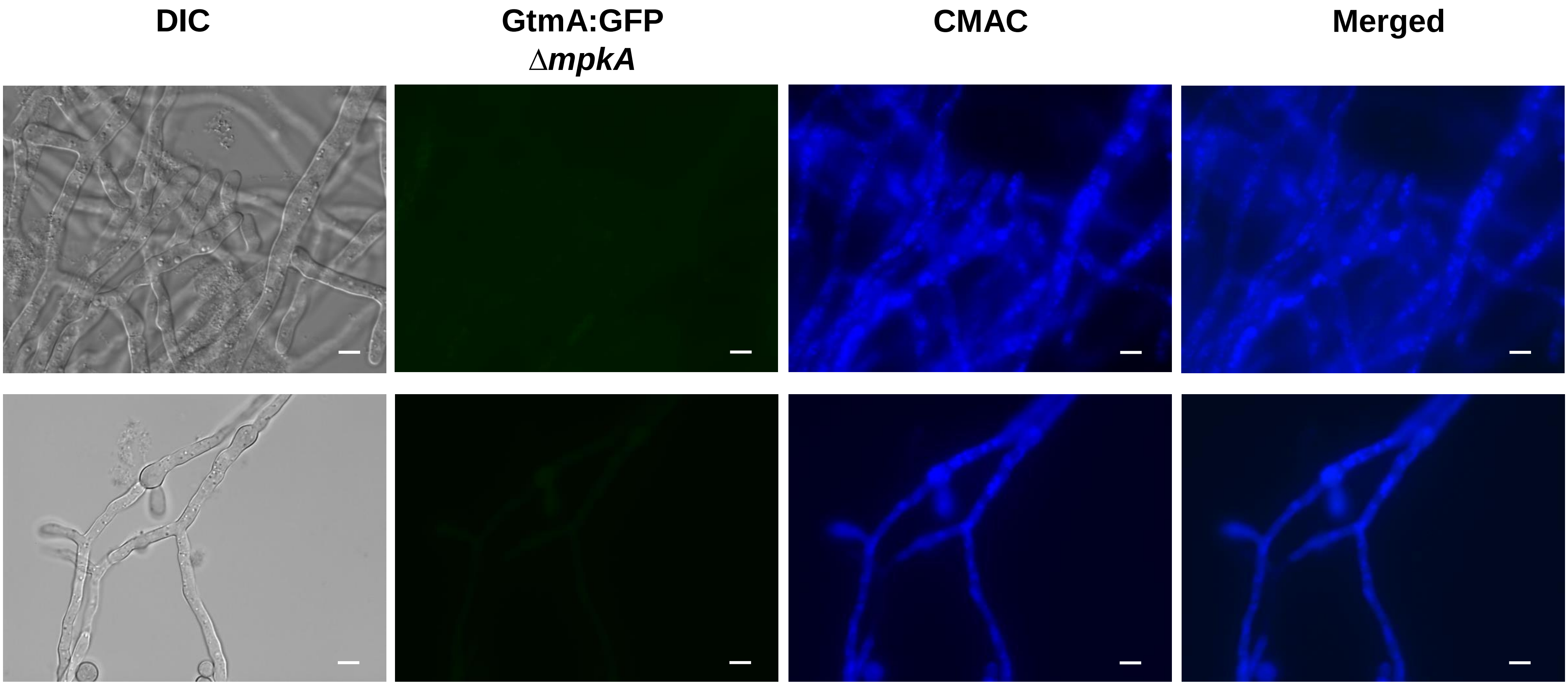

Supplementary Figure S2 – GliT:GFP and GtmA:GFP translocation to the vacuoles during 24 hs under gliotoxin production conditions in Czapek-dox medium. GliT:GFP and GtmA:GFP germlings were grown in liquid Czapek-dox medium for 24 h at 37 ºC. Representative brightfield, differential interference contrast (DIC), and Cell tracker Blue CMAC (CellTracker Blue CMAC Dye (7-amino-4-chloromethylcoumarin) for vacuolar staining.

Supplementary Figure S3 – *Aspergillus fumigatus* wild-type and mutants were grown on different stress conditions

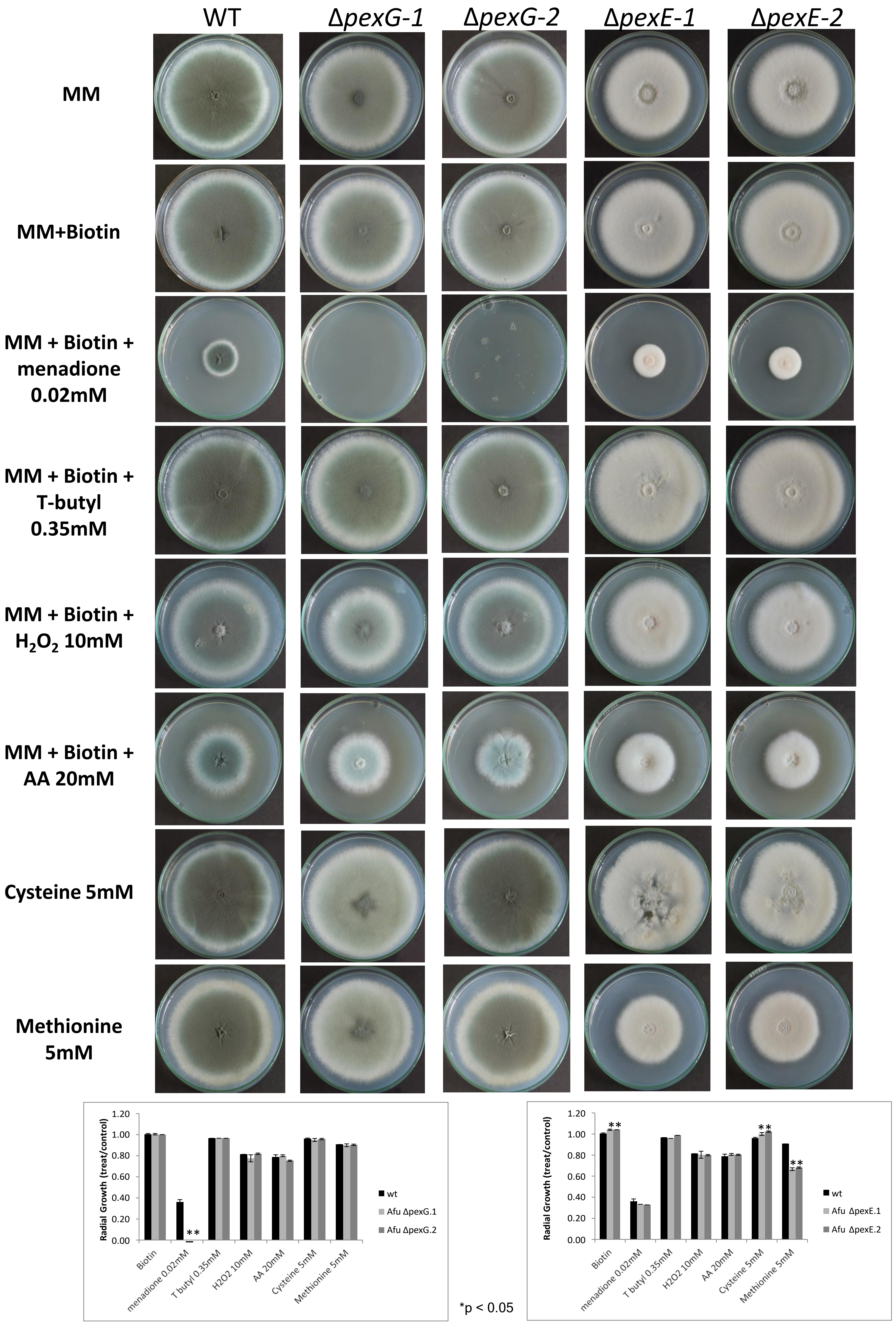

Supplementary Figure S3 – *Aspergillus fumigatus* wild-type and mutants were grown on different stress conditions. The *A. fumigatus* wild-type, Δ*pexE*, and Δ*pexG* strains were grown for 5 days at 37 ^{o}C on MM and MM with biotin, menadione, *t*-butyl hydroperoxide, allyl alcohol (AA), and hydrogen peroxide, or cysteine and methionine as single sulfur sources. The radial growth results are the mean of 3 independent experiments ± SD. ΔpexE an G mutants are compared to the wild-type (*, $p < 0.05$, *t*-test). Source data are provided as a Source Data file.

Supplementary Figure S4 – The Δ*pexG* mutant has increased *gli* genes and *gtmA* mRNA levels than the wild-type under non-inducing and inducing GT conditions.

A.

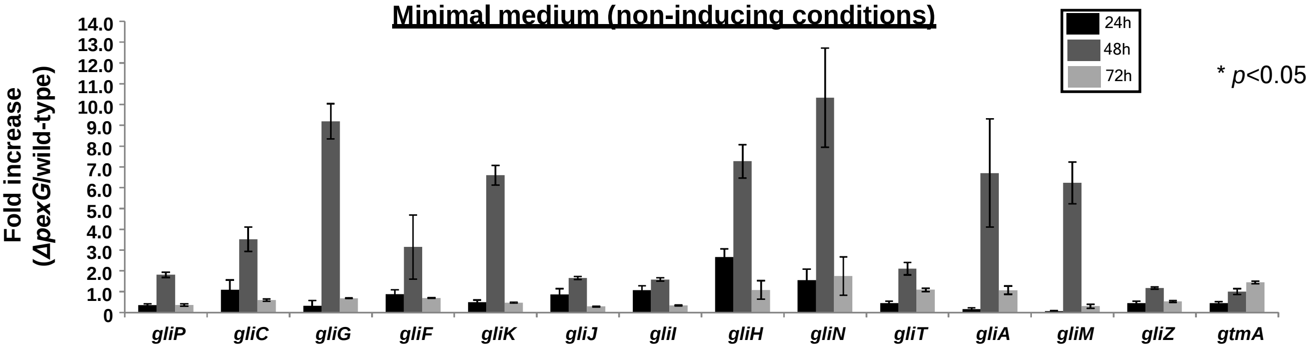

t test: Δ*pexG* versus Wild-type, * *p*<			.05		Minimal medium (nor		-inducing	conditic	ns)					
	gliP	***gliC***	***gliG***	***gliF***	***gliK***	***gliJ***	***gliI***	***gliH***	***gliN***	***gliT***	***gliA***	***gliM***	***gliZ***	***gtmA***
**24h**	*	no	*	*	*	no	no	*	*	*	*	*	*	*
**48h**	*	*	*	*	*	*	*	*	*	*	*	*	*	no
**72h**	*	*	*	*	*	*	*	no	no	no	no	*	*	*

B.

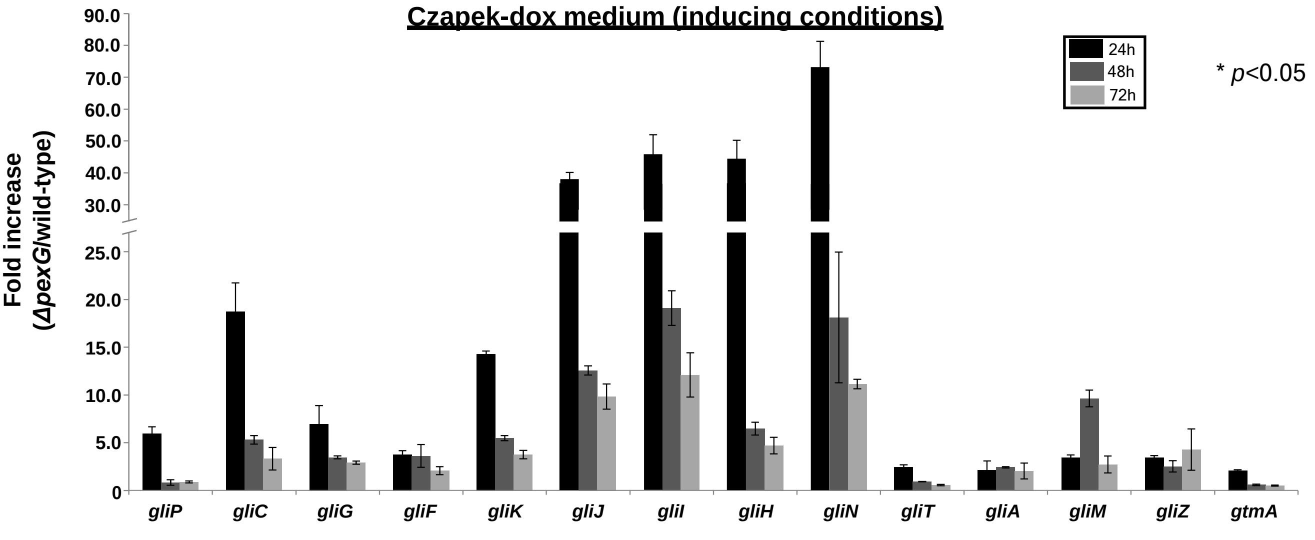

t test: Δ*pexG* versus Wild-type, * *p*<0.05					Czapek-dox medium (inducin			conditions						
	gliP	***gliC***	***gliG***	***gliF***	***gliK***	***gliJ***	***gliI***	***gliH***	***gliN***	***gliT***	***gliA***	***gliM***	***gliZ***	***gtmA***
**24h**	*	*	*	*	*	*	*	*	*	*	*	*	*	*
**48h**	no	*	*	*	*	*	*	*	*	*	*	*	*	*
**72h**	no	*	*	*	*	*	*	*	*	*	no	*	*	*

C.

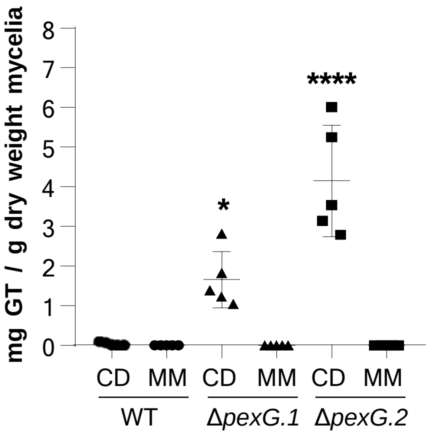

Ordinary one- way Anova; Dunnett's multiple comparisons test	Significant ?	Summary	Adjusted P Value
wt CD vs. Δ*pexG.1* CD	Yes	*	0.0287
wt CD vs. Δ*pexG.2* CD	Yes	****	<0.0001

Czapek Dox - 72hs			
**samples**	GT (mg)	Dry weight of the mycelia (g)	**GT (mg) / Dry weight (g)**
**WT**			
mean	0.013	0.281	**0.045**
SD	0.012	0.014	**0.042**
**Δ*pexG-1***			
mean	0.479	0.287	**1.660**
SD	0.212	0.009	**0.708**
pexG-2			
mean	1.182	0.284	**4.147**
SD	0.436	0.013	**1.406**

MM - 72hs			
**samples**	GT (mg)	Dry weight of the mycelia (g)	**GT (mg) / Dry weight (g)**
**WT**			
mean	0.000	0.262	**0.000**
SD	0.000	0.028	**0.000**
**Δ*pexG-1***			
mean	0.000	0.253	**0.000**
SD	0.000	0.026	**0.000**
**Δ*pexG-2***			
mean	0.000	0.256	**0.000**
SD	0.000	0.029	**0.000**

Supplementary Figure S4 – The Δ*pexG* mutant has increased *gli* genes and *gtmA* mRNA levels than the wild-type under non-inducing and inducing GT conditions. (a and b) RTqPCR of the *gli* genes and *gtmA* mRNA levels under non-inducing and inducing conditions with the *A. fumigatus* wild-type and Δ*pexG* mutant grown for 24, 48, and 72 h at 37 °C. Fold increase ratios equal to 1, below 1 or above 1 corresponded to similar, lower or higher mRNA levels in the Δ*pexG* mutant than in the wild-type. Statistical analysis was performed by using *t* test comparing Δ*pexG* versus Wild-type, * $p<0.05$. Results are the mean of 3 biologically independent experiments (with two technical replicates) ± SD. (c) Gliotoxin (GT) production. The results are the mean of n= 5 biological replicates ± SD. Source data are provided as a Source Data file.

Supplementary Figure S5 – Southern Blots and PCR schemes to check different constructions in *A. fumigatus and A. nidulans.*

A.

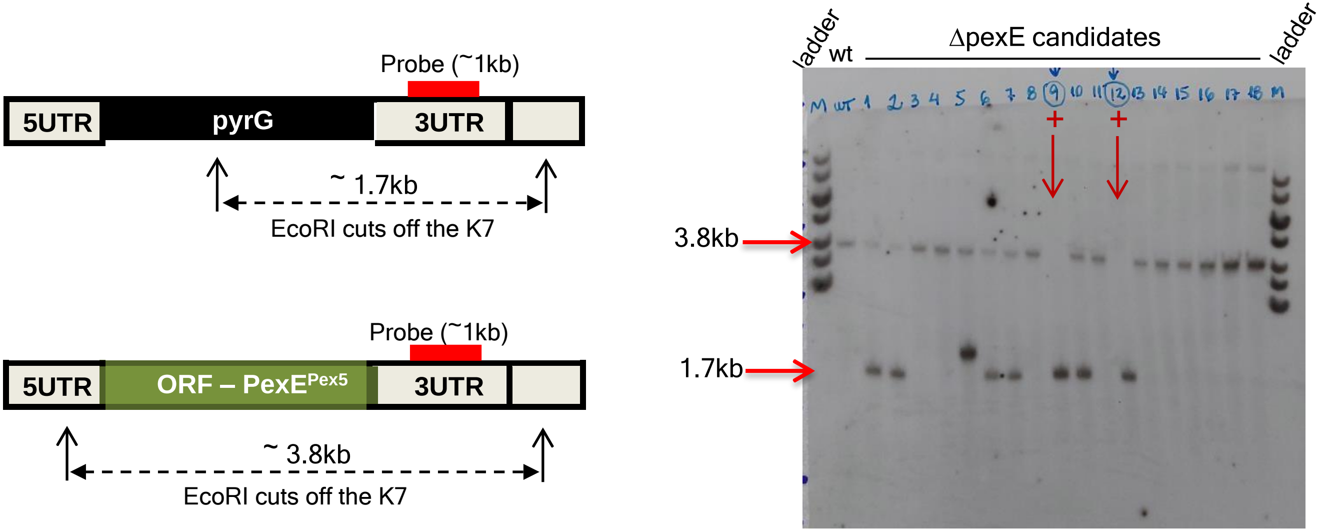

B.

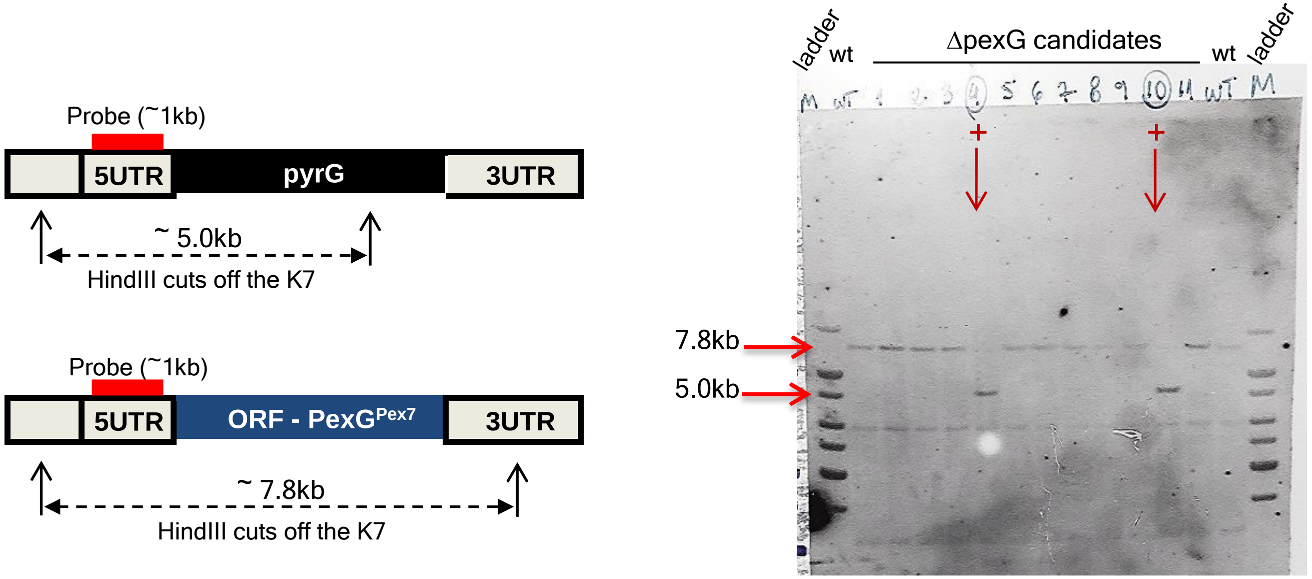

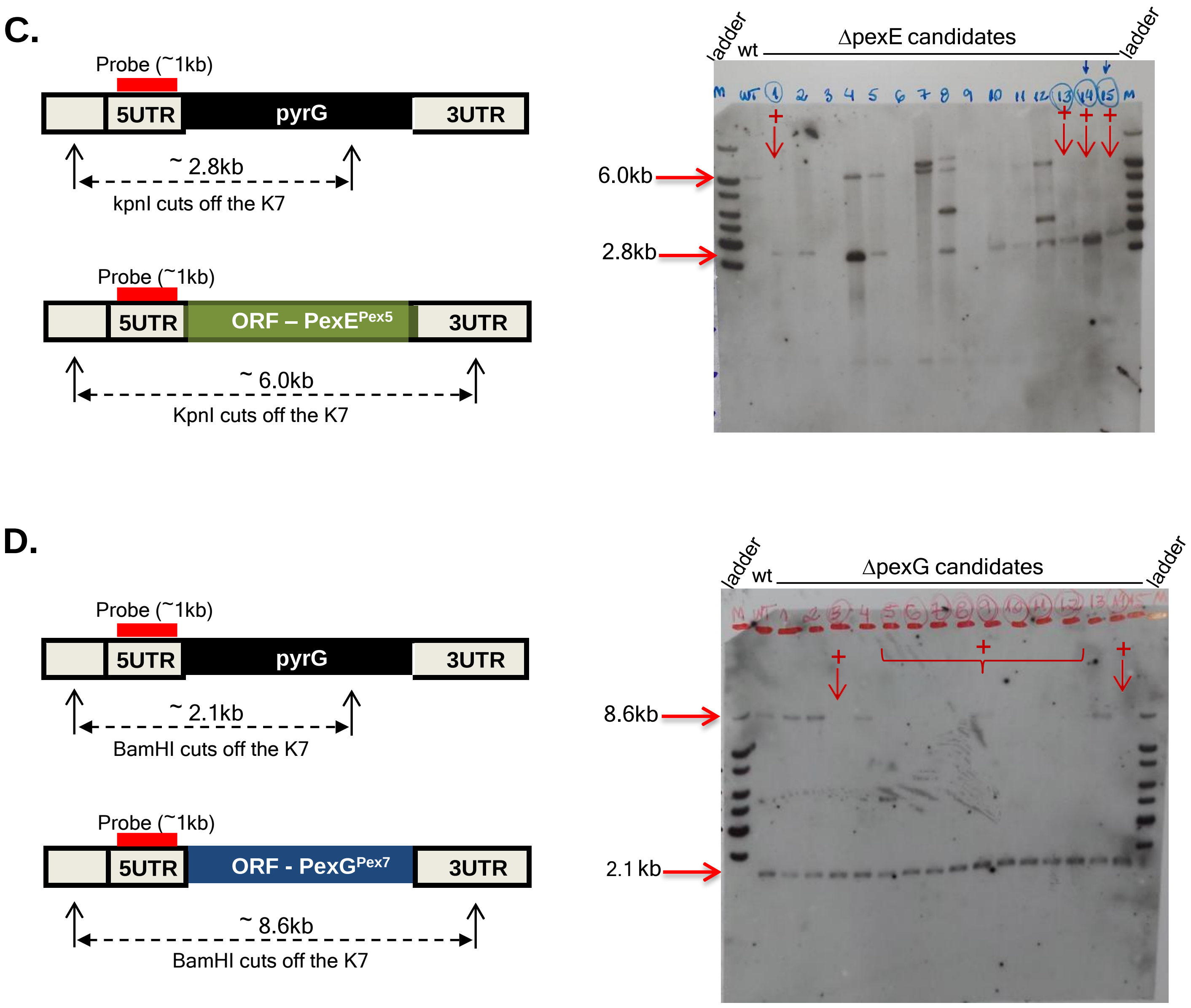
C.
Probe (~1kb)
5UTR
pyrG
3UTR
~ 2.8kb
kpnI cuts off the K7
Probe (~1kb)
5UTR
ORF – PexE^Pex5
3UTR
~ 6.0kb
KpnI cuts off the K7
ladder
wt
ΔpexE candidates
ladder
6.0kb
2.8kb
D.
Probe (~1kb)
5UTR
pyrG
3UTR
~ 2.1kb
BamHI cuts off the K7
Probe (~1kb)
5UTR
ORF - PexG^Pex7
3UTR
~ 8.6kb
BamHI cuts off the K7
ladder
wt
ΔpexG candidates
ladder
8.6kb
2.1 kb

E.

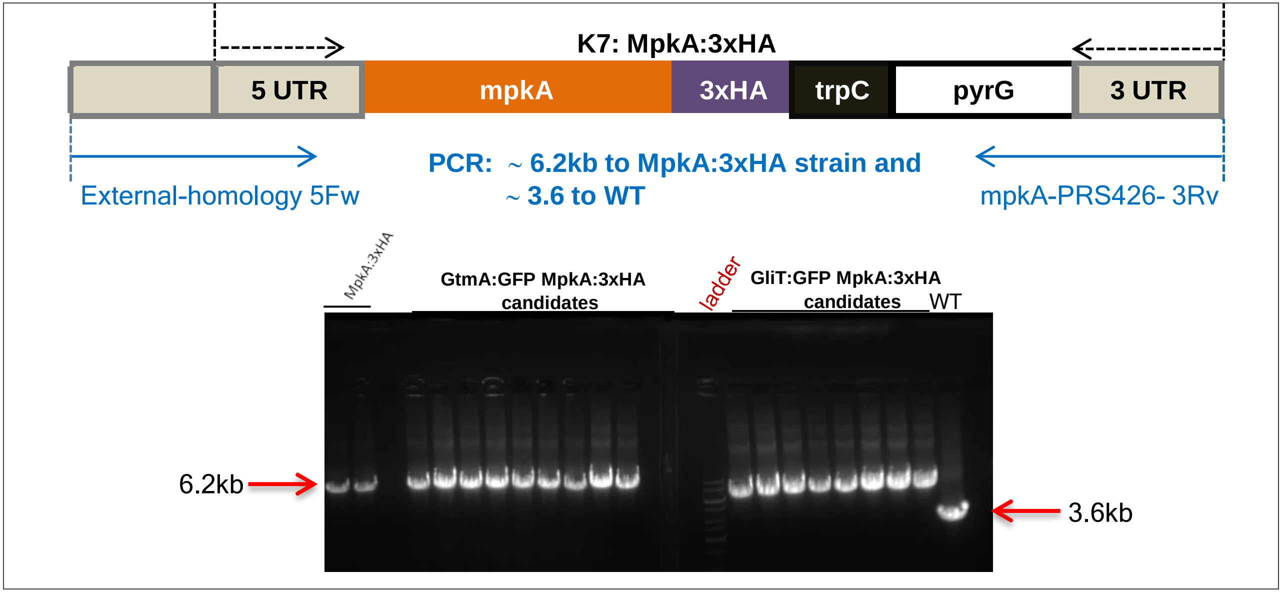
K7: MpkA:3xHA
5 UTR
mpkA
3xHA
trpC
pyrG
3 UTR
External-homology 5Fw
PCR: ~ 6.2kb to MpkA:3xHA strain and
~ 3.6 to WT
mpkA-PRS426- 3Rv
MpkA:3xHA
GtmA:GFP MpkA:3xHA
candidates
ladder
GliT:GFP MpkA:3xHA
candidates
WT
6.2kb
3.6kb

F.

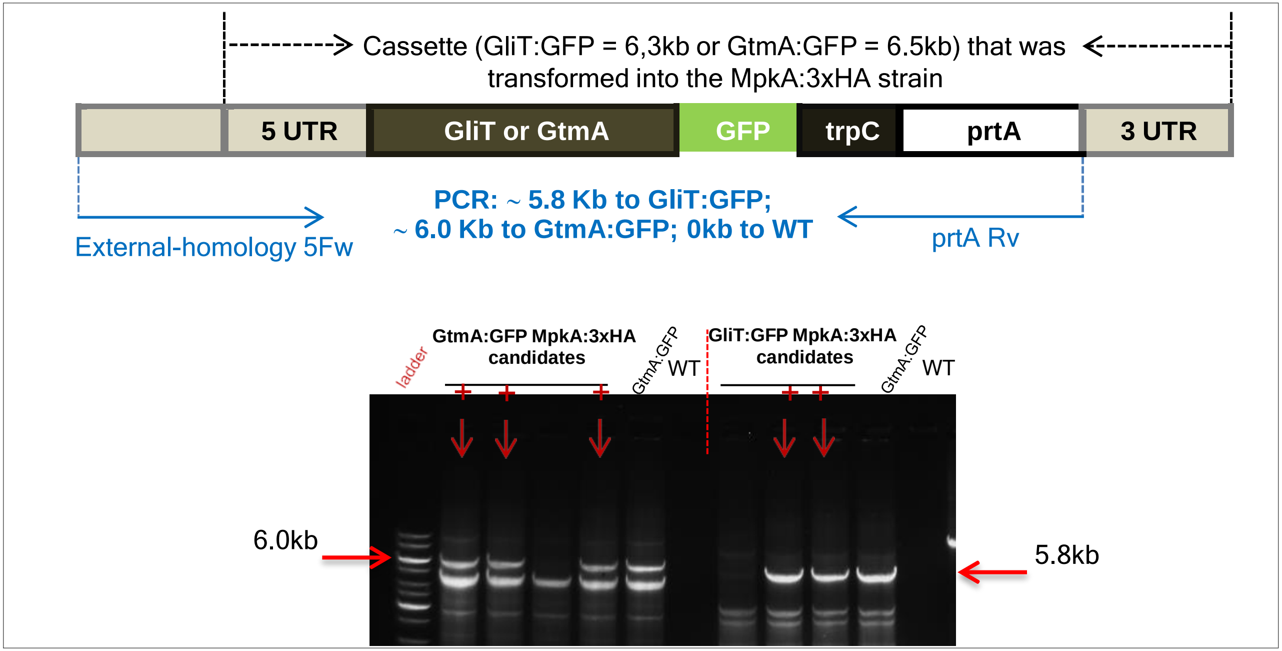
Cassette (GliT:GFP = 6,3kb or GtmA:GFP = 6.5kb) that was
transformed into the MpkA:3xHA strain
5 UTR
GliT or GtmA
GFP
trpC
prtA
3 UTR
PCR: ~ 5.8 Kb to GliT:GFP;
~ 6.0 Kb to GtmA:GFP; 0kb to WT
External-homology 5Fw
prtA Rv
ladder
GtmA:GFP MpkA:3xHA
candidates
GtmA:GFP
WT
GliT:GFP MpkA:3xHA
candidates
GtmA:GFP
WT
6.0kb
5.8kb

G.

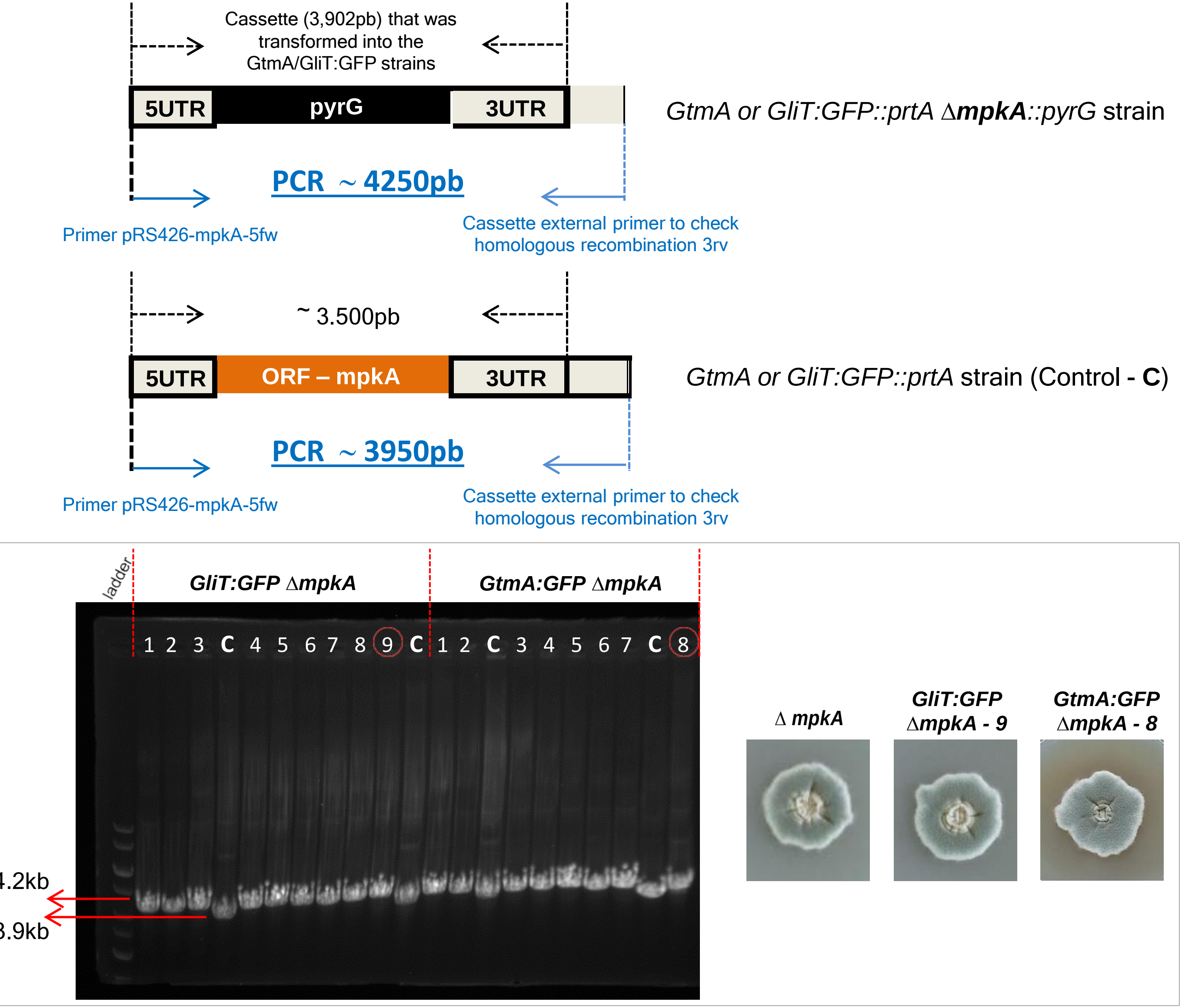

Supplementary Figure S5 – (A and B) Southern Blot scheme for *A. fumigatus ΔpexE* and *ΔpexG.* (C and D) Southern Blot scheme for *A. nidulans ΔpexE* and *ΔpexG.* (E) PCR scheme to check the MpkA:3xHA construction. (F) PCR scheme to check GliT:GFP and GtmA:GFP constructions. (G) PCR scheme to check the *GliT:GFP ΔmpkA* and GtmA:GFP *ΔmpkA* strains, and radial growth (MM, 5 days, 37℃).

Supplementary Table S1 - Protein kinase mutants

Gene	Description
AFUB_045810	Putative protein kinase
AFUB_030660	Serine threonine protein kinase, putative
AFUB_052450	Protein kinase, putative
AFUB_021710	pakA, Serine/threonine kinase Ste20
AFUB_053300	Protein kinase, putative
AFUB_012420	Mitogen-activated protein kinase (SakA)
AFUB_032300	Protein kinase, putative
AFUB_044560	Protein kinase, putative
AFUB_043130	MAP kinase kinase Ste7
AFUB_016170	Protein kinase (Lkh1), putative
AFUB_010510	kin1, predicted serine/threonine protein kinase
AFUB_018770	Non-specific serine/threonine protein kinase
AFUB_020560	Calcium/calmodulin-dependent protein kinase, putative
AFUB_029320	Calcium/calmodulin-dependent protein kinase, putative
AFUB_025560	Protein kinase (NpkA), putative
AFUB_038630	Protein serine/threonine kinase (Ran1), putative
AFUB_029240	Mitochondrial pyruvate dehydrogenase kinase, putative
AFUB_029820	Protein kinase, putative
AFUB_006780	schA, cAMP-dependent protein kinase-like, putative
AFUB_039620	Calcium/calmodulin dependent protein kinase, putative
AFUB_045840	Serine protein kinase, putative
AFUB_051750	Pyruvate dehydrogenase kinase, putative
AFUB_027640	Pyruvate dehydrogenase kinase
AFUB_038060	MAP kinase kinase kinase (Bck1), putative
AFUB_001600	Serine/threonine protein kinase, putative
AFUB_053500	Serine/threonine protein kinase, putative
AFUB_018600	Protein kinase, putative
AFUB_010360	MAP kinase kinase kinase SskB, putative
AFUB_044400	Uncharacterized protein
AFUB_035220	Ssn3, Cyclin-dependent protein kinase, putative
AFUB_019930	Serine/threonine protein kinase, putative
AFUB_017750	Protein kinase, putative
AFUB_027480	Protein kinase domain-containing protein
AFUB_030570	Protein kinase, putative
AFUB_053520	Calcium/calmodulin dependent protein kinase, putative
AFUB_039100	Serine/threonine protein kinase, putative
AFUB_007300	Serine/threonine protein kinase, putative
AFUB_014350	Serine/threonine protein kinase (Kin4), putative
AFUB_006320	Serine/threonine protein kinase, putative
AFUB_052630	Cyclin-dependent protein kinase PhoA
AFUB_011380	Protein kinase, putative
AFUB_071620	hypothetical protein
AFUB_056110	pkaC2, cAMP-dependent protein kinase catalytic subunit, putative

AFUB_082830	Serine/threonine protein kinase, putative
AFUB_027890	cAMP-dependent protein kinase catalytic subunit PkaC1
AFUB_056020	Serine/threonine protein kinase, putative
AFUB_059390	Protein kinase, putative
AFUB_059090	Serine/threonine protein kinase (Nrc-2), putative
AFUB_066150	atg1, putative Serine/threonine kinase
AFUB_090090	Serine/threonine protein kinase, putative
AFUB_078920	Casein kinase family protein
AFUB_077790	Protein kinase, putative
AFUB_078810	MpkB, Mitogen-activated protein kinase
AFUB_075210	Protein kinase, putative
AFUB_066030	Protein kinase, putative
AFUB_053960	MAP kinase kinase kinase Ste11/SteC
AFUB_070630	MpkA, Mitogen-activated protein kinase
AFUB_095720	Protein kinase, putative
AFUB_060320	Phosphotidylinositol kinase Tel1, putative
AFUB_087320	Protein kinase, putative
AFUB_079830	Uncharacterized protein
AFUB_078980	Protein kinase, putative
AFUB_056640	Mitogen-activated protein kinase
AFUB_074550	Protein kinase, putative
AFUB_071600	Serine/threonine-protein kinase
AFUB_087120	NACHT and Ankyrin domain protein
AFUB_075350	Uncharacterized protein
AFUB_054020	Serine/threonine protein kinase, putative
AFUB_081540	Protein kinase, putative
AFUB_055480	Serine/threonine protein kinase, putative
AFUB_098230	Inositol kinase kinase (UvsB), putative
AFUB_099170	Protein kinase Yak1, putative
AFUB_096030	Serine/threonine protein kinase (Kcc4), putative
AFUB_096080	Serine/threonine-protein kinase, putative
AFUB_099990	Serine protein kinase Sky1, putative
AFUB_006190	MAP kinase kinase (Mkk2), putative
AFUB_089280	Cell-cycle checkpoint protein kinase, putative
AFUB_101210	Sensor histidine kinase/response regulator, putative
AFUB_020650	Two-component osmosensing histidine kinase (Bos1), putative
AFUB_044260	Putative uncharacterized protein
AFUB_035990	Protein kinase domain-containing protein
AFUB_096660	Putative uncharacterized protein
AFUB_063270	Protein kinase Scy1, putative
AFUB_045550	Calcium/calmodulin dependent protein kinase, putative
AFUB_060950	Inositol hexaphosphate kinase KCS1, putative
AFUB_040390	Protein kinase domain-containing protein
AFUB_081220	Putative uncharacterized protein
AFUB_089460	Putative uncharacterized protein
AFUB_093800	Snf1 kinase complex beta-subunit Gal83, putative

AFUB_076300	Sensor histidine kinase/response regulator Fos-1
AFUB_075230	Sensor histidine kinase/response regulator, putative
AFUB_074100	Checkpoint protein kinase (SldA), putative
AFUB_067080	Putative uncharacterized protein
AFUB_001940	Sensor histidine kinase/response regulator, putative
AFUB_017740	Osmolarity two-component system protein sln1
AFUB_100220	Sensor histidine kinase/response regulator, putative
AFUB_036640	Sensor histidine kinase/response regulator, putative
AFUB_009070	Protein kinase family protein
AFUB_096590	Putative uncharacterized protein
AFUB_045710	Putative uncharacterized protein
AFUB_030290	Protein kinase domain-containing protein
AFUB_080040	Putative uncharacterized protein
AFUB_095050	Putative uncharacterized protein
AFUB_063830	Ubiquinone biosynthesis protein, putative
AFUB_101530	Sensor histidine kinase/response regulator, putative
AFUB_071990	N/A
AFUB_047210	Serine/threonine protein kinase, putative
AFUB_048440	Protein kinase, putative
AFUB_061120	Protein kinase domain-containing protein

Supplementary Table S1 - Protein kinases involved in gliotoxin self-defense. Screening in the presence of MM+GT compared to MM by quantifying radial growth susceptibility. For this screening, 109 PK mutants were grown on solid MM and MM+30 μg/mL of GT.

Supplementary Table S2 - Strains used in this work.

Strain	Genotype	Reference
A. fumigatus		
A1163	Δ*akuB pyrG*$^+$	[1]
CEA17	Δ*akuB pyrG*$^-$	[1]
Δ*rglT*	*MAT1-1*; *delta rglT::hph*	[2]
Δ*rglT*::*rglT*	*MAT1-1*; *delta rglT; delta rglT*::*rglT*	[3]
Δ*mpkA*	Δ*mpkA::hph*	[4]
Δ*mpkB*	Δ*mpkB::ptrA*; PT^R	[4]
Δ*mpkC*	Δ*mpkC::hph*	[5]
Δ*sakA* Δ*mpkC*	Δ*mpkC::prtA* Δ*sakA::hph*	[5]
Δ*sakA*	Δ*sakA::hph*	[5]
GtmA:GFP	*GtmA:GFP::hph*	[6]
GliT:GFP	*GliT:GFP::ble*	[7]
GtmA:GFP mpkA:3xHA	*GtmA:GFP::prtA; mpkA:3xHA::pyrG*	This work
GliT:GFP mpkA:3xHA	*GliT:GFP::prtA; mpkA:3xHA::pyrG*	This work
GtmA:GFP Δ*mpkA*	*GtmA:GFP::prtA;* Δ*mpkA::pyrG*	This work
GliT:GFP Δ*mpkA*	*GliT:GFP::prtA;* Δ*mpkA::pyrG*	This work
Δ*pex E1 and* Δ*pexE2*	Δ*pexE::pyrG*	This work
Δ*pex G1 and* Δ*pexG2*	Δ*pexE::pyrG*	This work
A. nidulans		
AGB551	*pyroA4, pyrG89,nkuAD::argB, veA+*	[8]
ΔAN1368 (Δ*rglT*)	*delta AN1368::pyrG; argB2 delta-nkuA::argB; pyroA4; veA1*	[3]
ΔAN1368::AN1368	*delta AN1368; delta AN1368::AN1368; argB2 delta-nkuA::argB; veA1*	[3]
Δ*pex E1 and* Δ*pexE2*	Δ*pexE::pyrG*	This work
Δ*pex G1 and* Δ*pexG2*	Δ*pexE::pyrG*	This work

Supplementary Table S3 - List of primers used in this work.

Primer	Sequence 5’- 3’
Afu5g06800 sybr fw	ACCTTGCTTGCAATCGACAG
Afu5g06800 sybr rv	TGCAGCAGAATGAGCAGGTAG
Afu8g07360 sybr fw	TTCGTGCACAGTTGGTTCTG
Afu8g07360 sybr rv	TTAATGAACGCGTGCGCATG
Afu5g03540 sybr fw	AAGTGTACACGCTCAACACG
Afu5g03540 sybr rv	TTCAGCACGATGTTGTACGC
Afu3g01750 sybr fw	ACACTCAGTCGCTACAACTACG
Afu3g01750 sybr rv	AACGCCGCAAAGAAGAGAAC
Afu6g10310 sybr fw	ATGCTGCGTATGCCAATGTC
Afu6g10310 sybr rv	TGCCATTGCACGCTATC
Afu1g10390 sybr fw	TTGGCGTGGCATTGTTGAAC
Afu1g10390 sybr rv	TCTGTGAAAGAGCGGATTCG
Afu6g12780 sybr fw	TTGCGGATAAGATGCATGGC
Afu6g12780 sybr rv	AACGCCATGTCAGGTTTGTC
Afu8g04630 sybr fw	TTGCCTTTGTTGCTGTCGTC
Afu8g04630 sybr rv	TAATAGTGGCCAGCGTGAACC
Afu5g01350 sybr fw	TGTTTATCGGTCCGTTTGCG
Afu5g01350 sybr rv	AGCACAAATCCCAGCAACAC
Afu7g065570 sybr fw	TTATTGCTGCGTTGGTGGTC
Afu7g065570 sybr rv	AGGAGGATCACGATCGCAATC
Afu_gliM sybr fw	CGATCTGTACCCCAACGAGT
Afu_gliM sybr rv	CCCAGGGTCTTCTGGAACTT
Afu_gliA sybr fw	TTTGCGATCAACGAACTCTG
Afu_gliA sybr rv	CCCTTGACGGACTGGAAGTA
Afu_gliH sybr fw	TTGCTGTCCTACCACGACAC
Afu_gliH sybr rv	CTTCTTCCGTTCCCGAATCT
Afu_gliG sybr fw	CTGATGTACATTGCCGATGC
Afu_gliG sybr rv	TTGTTTTGGGCAACTTCTCC
Afu_gliP sybr fw	CACAAGAGATGCGCCAGATA
Afu_gliP sybr rv	ATCATCGCCTCCATATCAGC
Afu_gliK sybr fw	CTCACGGCATACAGCGACTA
Afu_gliK sybr rv	ATAATCCAACCGAGCCACTG
Afu_gliC sybr fw	TTGTTTGGAGAGGTCCTTGG
Afu_gliC sybr rv	CCTTTGTTTTCGAGGCTCTG
Afu_gliN sybr fw	GTTCCACGTCCAGTCCATCT
Afu_gliN sybr rv	AGACTGCCTTCGGTGAATTG
Afu_gliI sybr fw	CCTTCCACCAGATTCCCTTT
Afu_gliI sybr rv	CGCTAGCACAATACCCCTGT
Afu_gliJ sybr fw	GTATCCGAGGAGGACGTCAA
Afu_gliJ sybr rv	CAACTTCCTCACCCGTTCTC
AfuPex5_pRS426_5UTR_fw **(P1)**	GTAACGCCAGGGTTTTCCCAGTCACGACGCA TTACTGATAACCGAGGATTGAG
AfuPex5 5UTR rv **(P2)**	CAGTGCCTCCTCTCAGACAGAATGGTTGACG AATTCACTAGAAACCTG
AfuPex5_3UTR_fw **(P3)**	GAGCATTGTTTGAGGCGAATTCGGATGGTAT CTCGTGCGCTTTC
AfuPex5_pRS426_3UTR_rv **(P4)**	GCGGATAACAATTTCACACAGGAAACAGCGC GTTTTTGACGGCGCCGGCGAC

AfuPex7_pRS426_5UTR_fw **(P5)**	GTAACGCCAGGGTTTTCCCAGTCACGACGCA ACTGGAGTAGTCCGCATCC
AfuPex7 5UTR rv **(P6)**	CAGTGCCTCCTCTCAGACAGAATGGTGCAAA ATACCGGGTATAGG
AfuPex7_3UTR_fw **(P7)**	GAGCATTGTTTGAGGCGAATTCCCAGTTCCAC CTACCCTGTATTGG
AfuPex7_pRS426_3UTR_rv **(P8)**	GCGGATAACAATTTCACACAGGAAACAGCGG GAAGACGCAGAAGCCAAGAG
AniPex5_pRS426_5UTR_fw **(P9)**	GTAACGCCAGGGTTTTCCCAGTCACGACGCA TTGGAAACCTCTTTTACGACG
AniPex5 5UTR rv **(P10)**	CAGTGCCTCCTCTCAGACAGAATGGTTGCGG GAAGAAGGTAAGTTC
AniPex5_3UTR_fw **(P11)**	GAGCATTGTTTGAGGCGAATTCGAAGGGTTA GGAGTCAGGCTGG
AniPex5_pRS426_3UTR_rv **(P12)**	GCGGATAACAATTTCACACAGGAAACAGCGT GTCTGGTCGACTAATTTCCG
AniPex7_pRS426_5UTR_fw **(P13)**	GTAACGCCAGGGTTTTCCCAGTCACGACGGT ACGTATAGTCATTCGCGTG
AniPex7 5UTR rv **(P14)**	CAGTGCCTCCTCTCAGACAGAATGGTCGAAG AAGGTTGAGTCACAG
AniPex7_3UTR_fw **(P15)**	GAGCATTGTTTGAGGCGAATTCGGCGTTCAG TTTCCCTTCCCC
AniPex7_pRS426_3UTR_rv **(P16)**	GCGGATAACAATTTCACACAGGAAACAGCGG ACCCAGTTGTAGGGGAAGGG
pyrG fw **(P17)**	GCATGCAAGCTTGGCGTATTCTGTCTGAGAG GAGGC
pyrG rv **(P18)**	GAATTCGCCTCAAACAATGCTCTTCACC
AfuMpkA_pRS426_5UTR_fw **(P19)**	GTAACGCCAGGGTTTTCCCAGTCACGACGTA CTCATTCCTTGTTCTGAT
AfuMpkA ORF rv **(P20)**	AGGAACATCATAGGGATAACCACCGCTACCA CCTCCCAGCGGGGGATGGATGTCCAA
AfuMpkA_3UTR_fw **(P21)**	ATCAGTGCCTCCTCTCAGACAGACGTTGACTT TCGTATGAAGC
AfuMpkA_pRS426_3UTR_rv **(P22)**	GCGGATAACAATTTCACACAGGAAACAGCGA AATCCGCTTCAACACCCCTTTC
OZG916	GGAGGTGGTAGCGGTGGT
OZG694	CTGTCTGAGAGGAGGCACTGAT
AfuGtmA_pRS426_5UTR_fw **(P23)**	GTAACGCCAGGGTTTTCCCAGTCACGACGGA CTGTACGCACAGCGTACGATTT
AfuGtmA ORF rv **(P24)**	CAATTGCCCGTCTGTCAGATCTTTGGTGAAGT TGAAAAGCGAGTA
AfuGtmA_3UTR_fw **(P25)**	CGGCTCATCGTCACCCCATGATAGCACAAAA TAAGCAGCTGTGGCTCG
AfuGtmA_pRS426_3UTR_rv **(P26)**	CGGATAACAATTTCACACAGGAAACAGCGTG ACAGTCCCGACGTTGCGAGTC
AfuGliT_pRS426_5UTR_fw **(P27)**	GTAACGCCAGGGTTTTCCCAGTCACGACGGT CCACGAGTTGGGCGGGACC
AfuGliT ORF rv **(P28)**	ACCACCGCTACCACCTCCTAGCTCCTGATCGA GACGAAA
AfuGliT_3UTR_fw **(P29)**	CGGCTCATCGTCACCCCATGATAGAAGTTTGT TATAGCTGTACAT
AfuGliT_pRS426_3UTR_rv **(P30)**	GCGGATAACAATTTCACACAGGAAACAGCGA ACTTCACCAGCCTGAGCCC

**P31**	GGAGGTGGTAGCGGTGGT
**P32**	CTGTCAGATCTGTAAAAAAGTTTCGGCCGGC
prtA FW **(P33)**	GATCTGACAGACGGGCAATTG
prtA RV **(P34)**	CTATCATGGGGTGACGATGAGCCG

References

1. Da Silva Ferreira ME, Kress MR, Savoldi M, Goldman MH, Härtl A, Heinekamp T, Brakhage AA, et al. The akuB(KU80) mutant deficient for nonhomologous end joining is a powerful tool for analysing pathogenicity in Aspergillus fumigatus. Eukaryot Cell. 2006; 5:207–211.

2. Furukawa T, van Rhijn N, Fraczek M, Gsaller F, Davies E, Carr P, et al. The negative cofactor 2 complex is a key regulator of drug resistance in Aspergillus fumigatus. Nat Commun. 2020;11. doi:10.1038/s41467-019-14191-1.

3. Ries LNA, Pardeshi L, Dong Z, Tan K, Steenwyk JL, Colabardini AC, et al. The Aspergillus fumigatus transcription factor RglT is important for gliotoxin biosynthesis and self-protection, and virulence. PLoS Pathog. 2020; Jul 15;16(7):e1008645. doi: 10.1371/journal.ppat.1008645.

4. V. Valiante, R. Jain, T. Heinekamp, A.A. Brakhage The MpkA MAP kinase module regulates cell wall integrity signaling and pyomelanin formation in Aspergillus fumigatus Fungal Genet. Biol., 46 (12) (2009), pp. 909-918

5. Bruder Nascimento AC, Dos Reis TF, de Castro PA, Hori JI, Bom VL, de Assis LJ, Ramalho LN, Rocha MC, Malavazi I, Brown NA, Valiante V, Brakhage AA, Hagiwara D, Goldman GH. Mitogen activated protein kinases SakA(HOG1) and MpkC collaborate for Aspergillus fumigatus virulence. Mol Microbiol. 2016 Jun;100(5):841-59. doi: 10.1111/mmi.13354. Epub 2016 Mar 23. PMID: 26878695.

6. Dolan SK, Bock T, Hering V, Owens RA, Jones GW, Blankenfeldt W, Doyle S. Structural, mechanistic and functional insight into gliotoxin bis-thiomethylation in Aspergillus fumigatus. Open Biol. 2017 Feb;7(2):160292. doi: 10.1098/rsob.160292. PMID: 28179499; PMCID: PMC5356443.

7. Schrettl M, Carberry S, Kavanagh K, Haas H, Jones GW, O'Brien J, Nolan A, Stephens J, Fenelon O, Doyle S. Self-protection against gliotoxin--a component of the gliotoxin biosynthetic cluster, GliT, completely protects Aspergillus fumigatus against exogenous gliotoxin. PLoS Pathog. 2010 Jun 10;6(6):e1000952. doi: 10.1371/journal.ppat.1000952. PMID: 20548963; PMCID: PMC2883607.

8. Bayram ,O .,Bayram ,O.S.,Ahm ed,Y.L.,M aruyam a,J.,Valerius,O.,Rizzoli,S.O .,Ficner, R.,Irniger,S.,Braus,G.H., 2012. The Aspergillus nidulans MAPK module AnSte11- Ste50-Ste7-Fus3 controls development and secondary metabolism .PLoS Genet. 8,e1002816.
